# HPV-driven oropharyngeal squamous cell cancer in Croatia — Demography and survival

**DOI:** 10.1371/journal.pone.0211577

**Published:** 2019-02-01

**Authors:** Ksenija Božinović, Ivan Sabol, Zoran Rakušić, Antonia Jakovčević, Mario Šekerija, Juraj Lukinović, Drago Prgomet, Magdalena Grce

**Affiliations:** 1 Division of Molecular Medicine, Ruđer Bošković Institute, Zagreb, Croatia; 2 Department of Radiation Oncology, Oncology Clinics, University Hospital Center Zagreb, Zagreb, Croatia; 3 School of Medicine, University of Zagreb, Zagreb, Croatia; 4 Clinical Department of Pathology and Cytology, University Hospital Center Zagreb, Zagreb, Croatia; 5 Croatian National Cancer Registry, Croatian Institute of Public Health, Zagreb, Croatia; 6 School of Medicine, Andrija Štampar School of Public Health, University of Zagreb, Zagreb, Croatia; 7 Clinic for Ear, Nose and Throat Diseases and Head and Neck Surgeries, University Hospital Center Zagreb, Zagreb, Croatia; University of Cincinnati College of Medicine, UNITED STATES

## Abstract

**Objectives:**

Head and neck squamous cell carcinoma (HNSCC) is the sixth most common malignancy worldwide. Main HNSCC risk factors are tobacco, alcohol, and high-risk human papillomavirus (HPV). HPV+ oropharyngeal squamous cell cancer (OPSCC) usually have different etiology, increasing incidence and often show an improved survival when compared to HPV-negative cases. The objective of the current study was to retrospectively examine the influence of HPV on the survival of OPSCC patients in a non-Western population setting.

**Materials and methods:**

We determined the presence of HPV DNA and/or RNA in 99 formalin-fixed paraffin embedded (FFPE) tissue samples of OPSCC patients treated between 2002 and 2015. Patients were compared based on laboratory, demographic, clinical, life style characteristics and survival.

**Results:**

HPV RNA was found in 29.3% cases. However, groups based on HPV data (either RNA, DNA or retrospectively collected p16 staining) did not show significant differences. Overall, 5-year survival was 30% with minimal influence of the HPV positivity.

**Conclusions:**

The HPV influence on survival of OPSCC patients is not identical between populations probably due to other factors overshadowing the HPV effect. This should be taken into account when treatment or policy decisions are made in each particular setting.

## Introduction

Head-and-neck cancer (HNC) is a group of malignancies that most commonly arise from the upper aerodigestive tract mucosa or lining of the head-and-neck regions [1]. It is the sixth most common malignancy worldwide. Most HNCs (95%) develop from squamous cell epithelia and are further characterized according to their primary site of origin. Most common sites are oral cavity, oropharynx, pharynx, larynx, and sinonasal tract [2]. Globally, head-and-neck squamous cell carcinoma (HNSCC) accounts for approximately 550,000 cases annually [3] and in Croatia, there were estimated 750 cases in 2014 [4].

The main risk factors in general for developing HNSCC are tobacco, alcohol use and the high-risk human papillomavirus (HPV) presence, with HPV-16 being found in majority of HPV associated HNSCC [5]. High-risk HPV is capable of transforming infected cells into cancerous ones by expressing oncoproteins E6 and E7, which bind, among others, to two of the most important tumor suppressor genes, p53 and pRB, respectively [5]. Previous studies have indicated that HNSCC, while very heterogeneous can be broadly divided into two groups: HPV positive (HPV+) and HPV negative (HPV–). Indeed, HPV is responsible for 30–50% HNSCC, with an increasing trend [5], found initially in the younger population of developed countries, although recent evidence suggests that HPV+ OPSCC is also increasing in older patients [6–8]. HPV+ HNSCC are often of oropharyngeal (OP) origin, with better prognosis and rare p53 mutations, usually with low levels of tobacco and alcohol use [5,9–11]. On the other hand, HPV–tumors are usually found in the elderly population with worse prognosis, without preferable origin, with frequent p53 mutations and having long history of tobacco and alcohol use. It is known that the incidence of HPV–HNSCC in the United States has been declining, presumably due to a reduced prevalence of tobacco smoking [12]. Over the past few decades, however, there has been a rise in HPV+ OPSCC [5,12–14]. In Croatia the trend between 1988 and 2008 was showing decreasing overall incidence of HNSCC [15], with more recent data corroborating the trend [4]. The proportion of HPV associated OPSCC is now approximately 70% in the developing countries, which is a substantial increase from previous rates [5,16]. Even though these two groups of HNSCC are etiologically different, the treatment remains the same [17,18]. However, there are indications that the treatment could be optimized for each group of patients, at least for HPV+ OPSCC patients [11,19]. Therefore, it is crucial to get a better understanding of the disease and get detailed information on patients including socio-epidemiological, clinical, biological and histopathological data.

In Croatia, only clinical guidelines for the treatment of laryngeal cancer currently exist, which is only a subset of HNSCC, accounting for ~33% of HNSCC cases in the country, while there are no guidelines for the treatment of cancers arising at other head-and-neck sites [15]. As HPV involvement seems to drive the increase of OPSCC incidence in developed countries, establishing guidelines for this cancer type could be of a future benefit to patients, but the baseline data for such guidelines are scarce. Thus, in this study, we assessed archival formalin-fixed paraffin embedded (FFPE) tissue of OPSCC patients for HPV presence (DNA) and activity (mRNA) to determine differences in patient characteristics. Furthermore, medical records together with Croatian National Cancer Registry data were evaluated to determine the influence of HPV on survival. The study objective was to gain a better understanding of OPSCC in Croatian and similar non-Western populations.

## Materials and methods

### Patient samples

For this study, we collected a subset of available FFPE tumor tissue from 104 OPSCC patients treated at the University Hospital Center Zagreb between 2002 and 2015. According to the International Classification of Disease (ICD-10), tumors included tonsils (C09), base of tongue (C01), soft palate (C05.1), lateral wall (C10.2) and posterior wall of oropharynx (C10.3). One patient had unspecified oropharynx tumor localization. Upon medical records reexamination, five cases were excluded as the tumor originated from supraglotis/hypopharynx, thus 99 cases were retained for analysis. The study was approved by the Bioethical Board of the Ruđer Bošković Institute (BEP-3748/2-2014) and the Ethical Board of the University Hospital Center Zagreb (8.1-14/47-2, 02/21-JG). Since the study was retrospective and performed on anonymized data from often deceased patients, the Ethical boards waived the necessity of obtaining additional informed consent. Medical records and patient’s vital status, including previous p16 staining where available, were obtained from the hospital information system and the Croatian National Cancer Registry [4] and contained relevant data collected during the establishment of the initial diagnosis including pathological assessment. Original patient management treatment decisions were not based upon HPV DNA or RNA testing but were made according to tumor stage and overall patient state.

Data from the National Cancer Registry indicates that in the same period there were approximately 1600 OPSCC patients diagnosed throughout Croatia with the same diagnosis codes as used in the manuscript. However, of this number almost 500 were reported to the Registry as C10.9 oropharynx, unspecified, which are underrepresented in our analyzed tissues. The University Hospital Center Zagreb treated 403 oropharyngeal cancer patients during this period. Thus, our sample represents approximately 7% of cancer patients in Croatia and approximately 25% of patients treated at the hospital.

Malignancies were staged according to the 7^th^ Edition of the American Joint Committee on Cancer (AJCC) and the Union for International Cancer Control (UICC) TNM Classification of Malignant tumors [20]. The pathologic classification was used and supplemented with the clinical classification if missing. For the survival analyses, both 7^th^ and 8^th^ AJCC Edition staging guidelines [20,21] have been used and compared. Patient survival time in months was calculated from the date of earliest diagnosis to registered time of death (all cause) or December 31^st^, 2017.

### Nucleic acid isolation

Approximately 5–7 10 μm serial sections of each tissue block were obtained for DNA and another 5–7 sections for RNA isolation. Precautions were taken to avoid sample cross-contamination, including change of knife and meticulous cleaning of the microtome for each new block as well as discarding top layers of each block that could potentially have been contaminated during storage. DNA was isolated with a NucleoSpin Tissue kit (Machery-Nagel), while RNeasy FFPE Kit (Qiagen) was used for total RNA isolation of HPV+ samples. Isolation procedures were performed according to the respective manufacturer’s protocol. Extracted DNA and RNA were quantified using NanoPhotometer (Implen, Germany).

### HPV DNA detection

Extracted DNA adequacy was further confirmed by PCR amplification of beta-actin gene (~99 bp) [22]. HPV DNA detection was performed using short primers suitable for FFPE tissue analysis, GP5/6 (~142bp amplicon) and SPF-10 (~65bp amplicon) previously described [23,24]. CaSki cell line DNA containing HPV-16 was used as positive control, while negative control reactions contained all reagents except DNA. All standard precautions for avoiding cross contamination were followed and reactions were prepared within a UV decontaminated laminar flow hood. PCR products (10 μl) were visualized after 3% agarose gel electrophoresis. A sample was considered as HPV DNA positive if consensus primers, either GP or SPF directed PCR was positive and the results valid if beta-actin directed PCR (internal control) was successful. HPV specific genotyping was not performed due to DNA degradation in FFPE samples.

### E6/E6*I mRNA expression analyses

All HPV DNA positive samples were selected for RNA isolation and HPV-16 E6 mRNA detection. RNAse-free DNAse digestion was performed to further limit DNA contamination. Briefly, 1 μg of RNA was reverse transcribed using QuantiTect Reverse Transcription kit (Qiagen) according to the manufacturer’s protocol. Most abundant splice variant of the E6 open reading frame (E6*I) was detected by PCR [25] and the amplicons (~86 bp) visualized by a 3% agarose electrophoresis. Suitability of cDNA for amplification was confirmed by beta-actin PCR (~99 bp). CaSki cell line cDNA served as positive control, while negative control contained all reagents except cDNA.

### Immunohistochemistry

The p16 data was obtained from medical records of staining done by the CINtec p16INK4a Histology Kit (mtm laboratories, Heidelberg, Germany) according to the manufacturer instructions [26]. The staining was evaluated by the same pathologist (AJ) for all patients. The overexpressed p16 (≥70%) in tumour cells was considered as positive.

### Statistical analyses

Based on HPV DNA and RNA testing results, samples were grouped in three distinct groups: HPV DNA/RNA negative), HPV DNA positive and HPV RNA positive. Statistical analyses were performed using R (v3.4.2) and MedCalc (v11.4 MedCalc Software, Ostend, Belgium). Patient’s characteristics for specific variables (including gender, tumor localization, grade, TNM and therapy) were compared between the groups using Test of Independence/Chi-Square and correlation. Age at diagnosis was analyzed using T-test. For statistical purposes, missing data was recoded as “Unknown” but retained to minimize possible selection bias. Survival data was assessed in MedCalc using Kaplan-Meier analyses from which all non-primary tumors (n = 8) and patients with unavailable survival data (n = 10) were excluded. When assessing tumor staging, patients were grouped to reduce the number of subgroups: for T classification “1–2” and “2–3” groups were considered. For N classification, “0–1” and “2–3” groups were made, while for tumor stages “I-II” and “III-IV” groups were considered irrespective of staging edition. Patients self-reporting weekly or more common strong spirit use or daily other alcohol use were grouped as heavy drinkers, while patients with self-reported occasional or limited alcohol use were grouped as occasional drinkers. Influence of individual variables on survival were modeled with Cox proportional hazards regression in univariate analysis for age, gender, HPV DNA positivity, HPV RNA positivity, p16 positivity, T classification, N classification, stage, grade, smoking history and drinking history. Several multivariate Cox proportional hazards regression models were created from selected significant or relevant variables (T/N classification) and HPV groups. Several models were created from all entered variables without removal of non-significant variables to enable the comparison of individual variable effects. However, the models including “Unknown” groups couldn’t be solved so for multivariate analysis, samples with “Unknown” factors were removed. *P* values less than 0.05 were considered significant.

## Results

**Fig 1:**
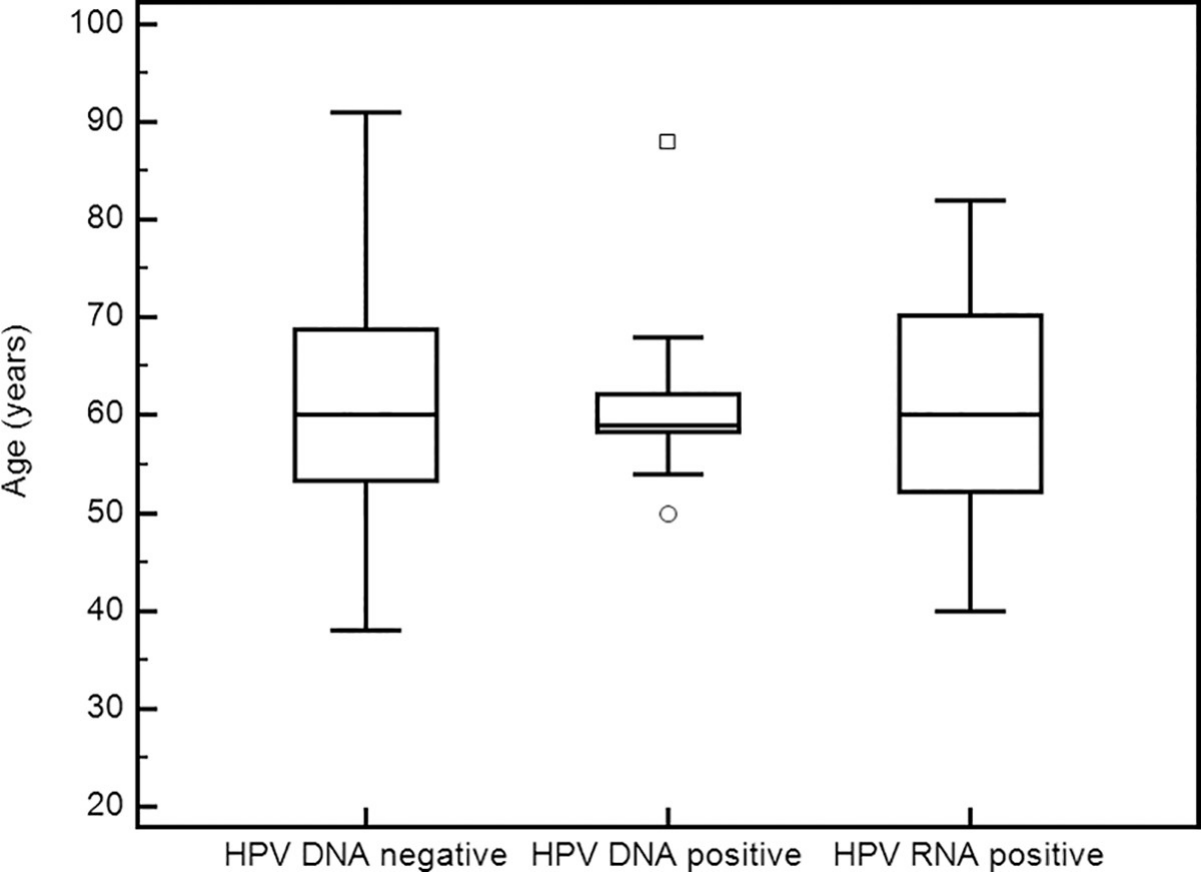
Age at diagnosis in sample groups. DNA positive group consists of samples tested positive for DNA and negative for RNA, while the RNA positive group tested positive for both DNA and RNA.

From a total of 99 OPSCC patients, 59 (59.6%) were HPV DNA negative and 40 (40.4%) HPV DNA positive, of which 11 (27.5%) were RNA negative, and 29 (72.5%) RNA positive. Statistical analysis was done on those 3 subgroups: HPV DNA/RNA negative (N = 59), DNA positive/RNA negative (N = 11), and DNA/RNA positive (N = 29). In addition, the tests were also performed on the whole HPV DNA positive subgroup, including both RNA negative and RNA positive samples (N = 40), but no additional statistical significance was found. Patient’s characteristics are presented in Table 1. Male patients were predominant in all groups, and account for 82.8% cases. Tonsil and base of tongue were the most common subsites, together accounting for more than 86% of cases. There were no statistically significant differences between the HPV groups in terms of age at the time of diagnosis (Fig 1) or gender. There were 14% never smokers and 13% never drinkers without significant differences between groups. There was no correlation between HPV and p16 results with Pearson’s correlation coefficient r = 0.14 for comparison with HPV RNA. The p16 positive staining was weakly statistically associated with patient’s older age (t-test, *P* = 0.042) and with cancer higher grade (Mann-Whitney, *P* = 0.017).

**Table 1:** Characteristics of the study population.

Variable		HPV DNA and RNA positive (N = 29)	HPV DNA positive and RNA negative (n = 11)	HPV negative (n = 59)	Total (n = 99) ^a^
**Gender**	Male	22 (26.8%)	10 (12.2%)	50 (60.9%)	82 (82.8%)
	Female	7 (41.2%)	1 (5.9%)	9 (52.9%)	17 (17.2%)
**Tumor site**	Tonsil	17 (32.1%)	6 (11.3%)	30 (56.6%)	53 (53.5%)
	Base of tongue	10 (30.3%)	5 (15.2%)	18 (54.6%)	33 (33.3%)
	Soft palate	0 (0%)	0 (0%)	7 (100%)	7 (7.1%)
	Lateral pharyngeal wall	2 (40%)	0 (0%)	3 (60%)	5 (5.1%)
	Oropharynx (unspecified)	0 (0%)	0 (0%)	1 (100%)	1 (1.0%)
**Age group**	<45	2 (33.3%)	0 (0%)	4 (66. 7%)	6 (6.1%)
	45–64	17 (27.9%)	9 (14.8%)	35 (57.4%)	61 (61.6%)
	65+	10 (31.3%)	2 (6.3%)	20 (62.5%)	32 (32.3%)
	Median age	60	59	60	60
**Smoking**	Active	4 (18.2%)	4 (18.2%)	14 (63.6%)	22 (22.2%)
	Former	2 (20%)	0 (0%)	8 (80%)	10 (10.1%)
	Does not smoke	6 (42.9%)	2 (14.3%)	6 (42.9%)	14 (14.1%)
	Unknown	17 (32.1%)	5 (9.4%)	31 (58.5%)	53 (53.5%)
**Drinking**	Heavy	1 (12.5%)	1 (12.5%)	6 (75%)	8 (8.1%)
	Occasional	5 (20%)	4 (16%)	16 (64%)	25 (25.3%)
	Does not drink	6 (46.6%)	1 (7.7%)	6 (46.2%)	13 (13.1%)
	Unknown	17 (32.1%)	5 (9.4%)	31 (58.5%)	53 (53.5%)

^a^total column percentages are calculated against the grand total

Clinical parameters are presented in Table 2; the majority of all patients with available data were diagnosed with stage III or worse cancer (76.6%) according to the 7^th^ edition AJCC guidelines [20], and there was no significant difference between groups. However, according to the 8^th^ edition AJCC guidelines [21], the majority of RNA positive tumors were staged as early and in this case the difference vs HPV–tumor group was significant (Chi-square test, P<0.0001). Only 10% patients, for which data were available, were treated non-surgically. Palliative therapy only was administered for 5 patients.

**Table 2:** Patient clinical characteristics.

Variable ^a^		HPV DNA and RNA positive (N = 29)	HPV DNA positive and RNA negative (n = 11)	HPV negative (n = 59)	Total ^b^ (n = 99)
**T**	1	4 (13.8%)	2 (18.2%)	5 (8.5%)	11 (11.1%)
	2	6 (20.7%)	4 (36.4%)	19 (32.2%)	29 (29.3%)
	3	6 (20.7%)	2 (18.2%)	8 (13.6%)	16 (16.2%)
	4	3 (10.3%)	1 (9.1%)	13 (22.0%)	17 (17.2%)
	Unknown	10 (34.5%)	2 (18.2%)	14 (23.7%)	26 (26.3%)
**N**	0	3 (10.3%)	3 (27.3%)	18 (30.5%)	24 (24.2%)
	1	5 (17.2%)	3 (27.3%)	4 (6.8%)	12 (12.1%)
	2	11 (37.9%)	3 (27.3%)	18 (30.5%)	32 (32.3%)
	3	0 (0%)	0 (0%)	5 (8.5%)	5 (5.1%)
	Unknown	10 (34.5%)	2 (18.2%)	14 (23.7%)	26 (26.3%)
**7**^**th**^ **edition staging guidelines**	Early	3 (10.3%)	2 (18.2%)	13 (22.0%)	18 (18.2%)
	I	1 (3.5%)	1 (9.1%)	3 (5.1%)	5 (5.1%)
	II	2 (6.9%)	1 (9.1%)	10 (16.9%)	13 (13.1%)
	Late	16 (55.2%)	7 (63.6%)	36 (61.0%)	59 (59.6%)
	III	6 (20.7%)	3 (27.3%)	7 (11.9%)	16 (16.2%)
	IVa	10 (34.5%)	4 (36.4%)	19 (32.2%)	33 (33.3%)
	IVb	0 (0%)	0 (0%)	5 (8.5%)	5 (5.1%)
	IVc	0 (0%)	0 (0%)	5 (8.5%)	5 (5.1%)
	Unknown	10 (34.5%)	2 (18.2%)	10 (16.9%)	22 (22.2%)
**8**^**th**^ **edition staging guidelines**	Early	16 (55.2%)	2 (18.2%)	13 (22.0%)	31 (31.3%)
	I	7 (24.1%)	1 (9.1%)	3 (5.1%)	11 (11.1%)
	II	9 (31.0%)	1 (9.1%)	10 (16.9%)	20 (20.2%)
	Late	3 (10.3%)	7 (63.6%)	36 (61.0%)	46 (46.5%)
	III	3 (10.3%)	3 (27.3%)	7 (11.9%)	13 (13.1%)
	IVa	0 (0%)	4 (36.4%)	19 (32.2%)	23 (23.2%)
	IVb	0 (0%)	0 (0%)	5 (8.5%)	5 (5.1%)
	IVc	0 (0%)	0 (0%)	5 (8.5%)	5 (5.1%)
	Unknown	10 (34.5%)	2 (18.2%)	10 (16.9%)	22 (22.2%)
**Grade**	1	9 (31.0%)	2 (18.2%)	13 (22.0%)	24 (24.2%)
	2	7 (24.1%)	6 (54.6%)	22 (37.3%)	35 (35.4%)
	3	13 (44.8%)	3 (27.3%)	21 (35.6%)	37 (37.4%)
	Unknown	0 (0%)	0 (0%)	3 (5.1%)	3 (3.0%)
**Therapy** ^c^	Surgical	18 (62.1%)	8 (72.7%)	39 (66.1%)	65 (65.7%)
	Surgery	11 (37.9%)	2 (18.2%)	16 (27.1%)	29 (29.3%)
	Surgery + RT	4 (13.8%)	5 (45.5%)	14 (23.7%)	23 (23.2%)
	Surgery + CRT	3 (10.314%)	1 (9.1%)	9 (15.3%)	13 (13.1%)
	Non-surgical	1 (3.5%)	1 (9.1%)	6 (10.2%)	8 (8.1%)
	Palliative	2 (6.9%)	0 (0%)	3 (5.1%)	5 (5.1%)
	Unknown	8 (27.6%)	2 (18.2%)	11 (18.6%)	21 (21.2%)
**p16**	Positive	7 (24.1%)	2 (18.2%)	18 (30.5%)	27 (27.3%)
	Negative	12 (41.4%)	7 (63.6%)	24 (40.7%)	43 (43.4%)
	Unknown	10 (34.5%)	2 (18.2%)	17 (28.8%)	29 (29.3%)

^a^ 26 patients had missing data for T/N classification of which 4 had recorded distant metastases and were thus classified as stage IVc

^b^ total percentages are calculated against grand total

^c^ RT- radio therapy, CRT–chemo and/or radiotherapy, non-surgical—any combination of chemo/radio therapy

**Fig 2:**
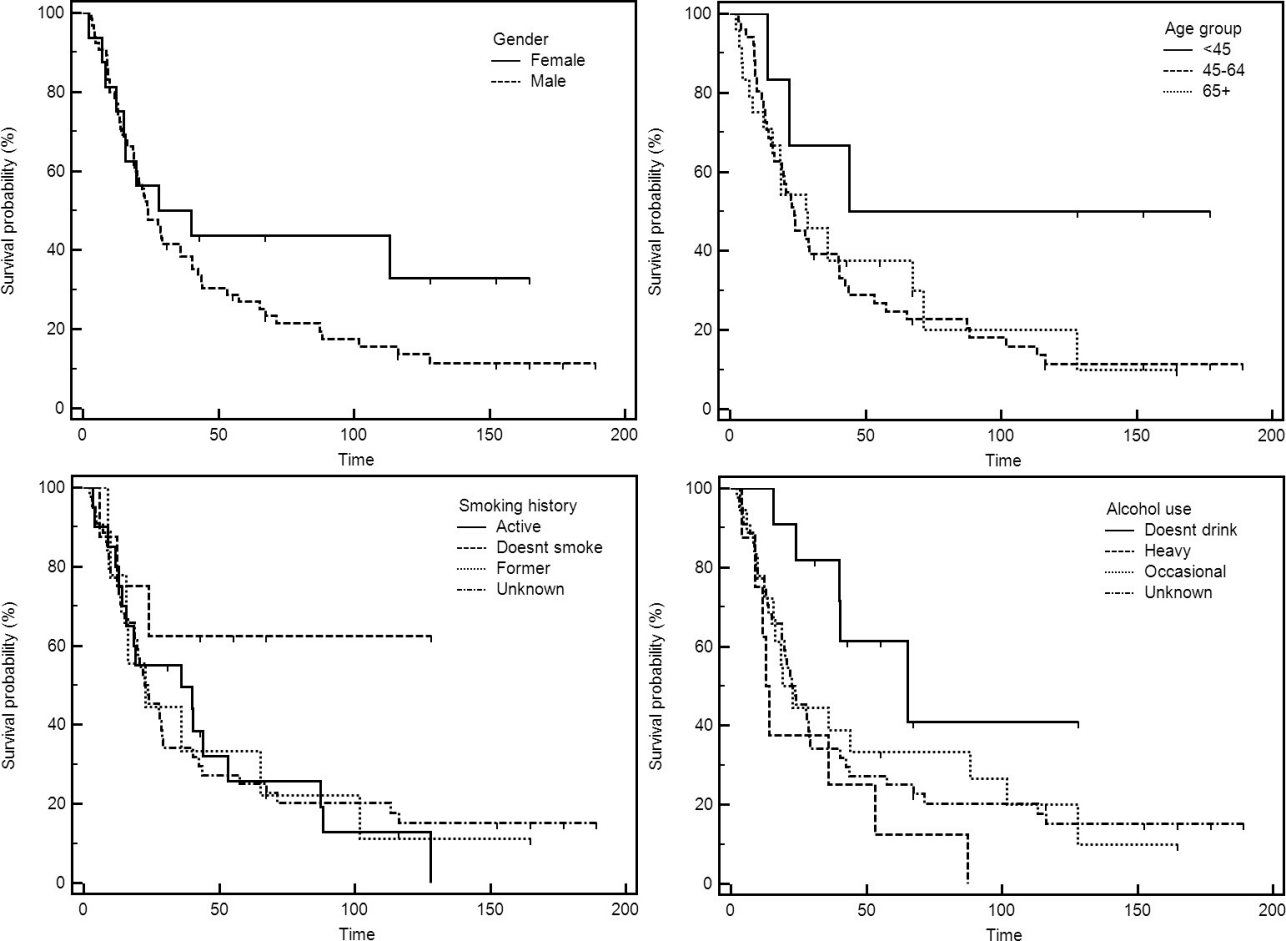
Overall survival of OPSCC patients based on the gender, age group at the time of diagnosis, smoking history and alcohol use.

**Fig 3:**
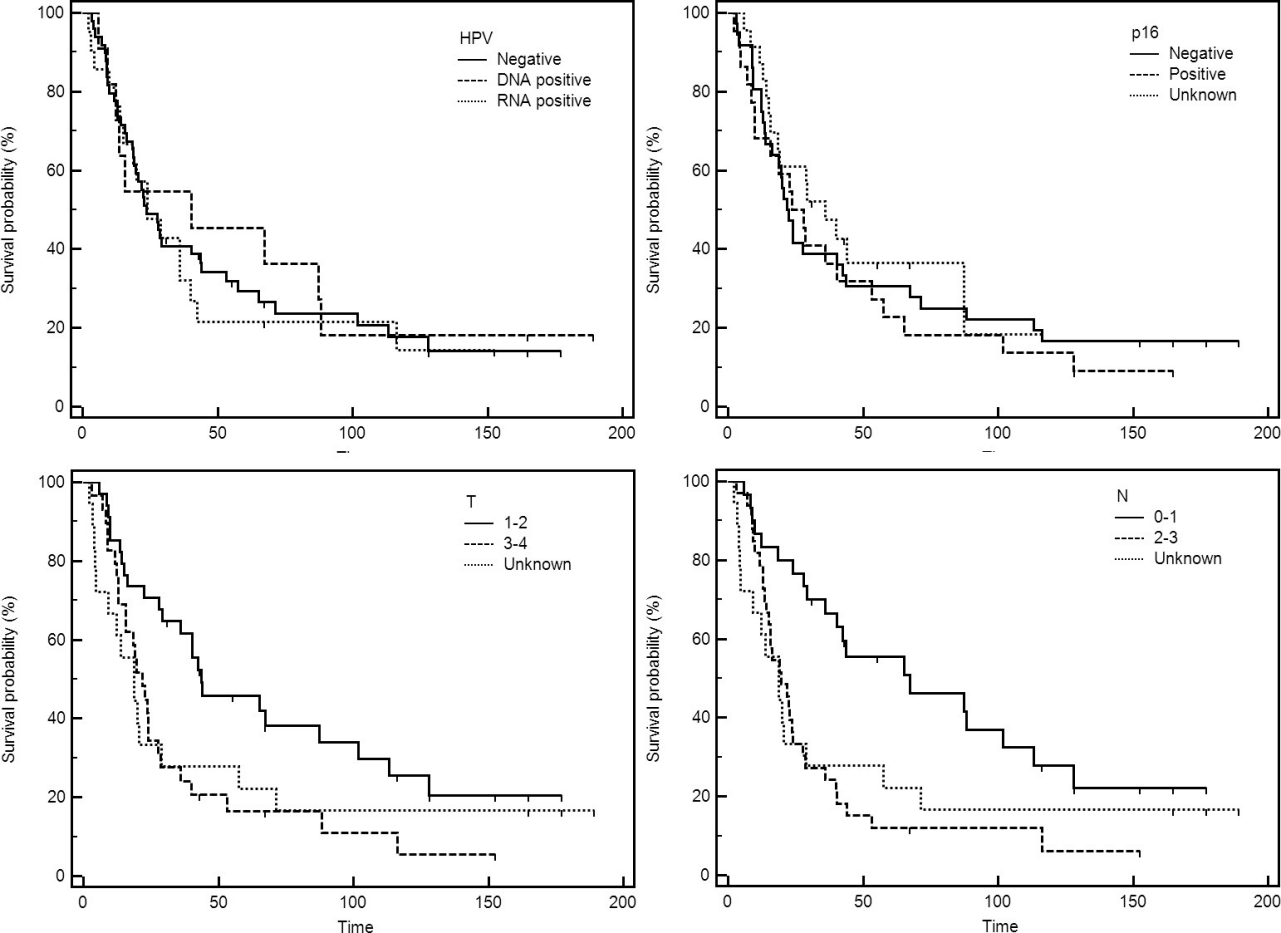
Overall survival of OPSCC patients based on HPV positivity, p16 data, and T and N classification.

**Fig 4:**
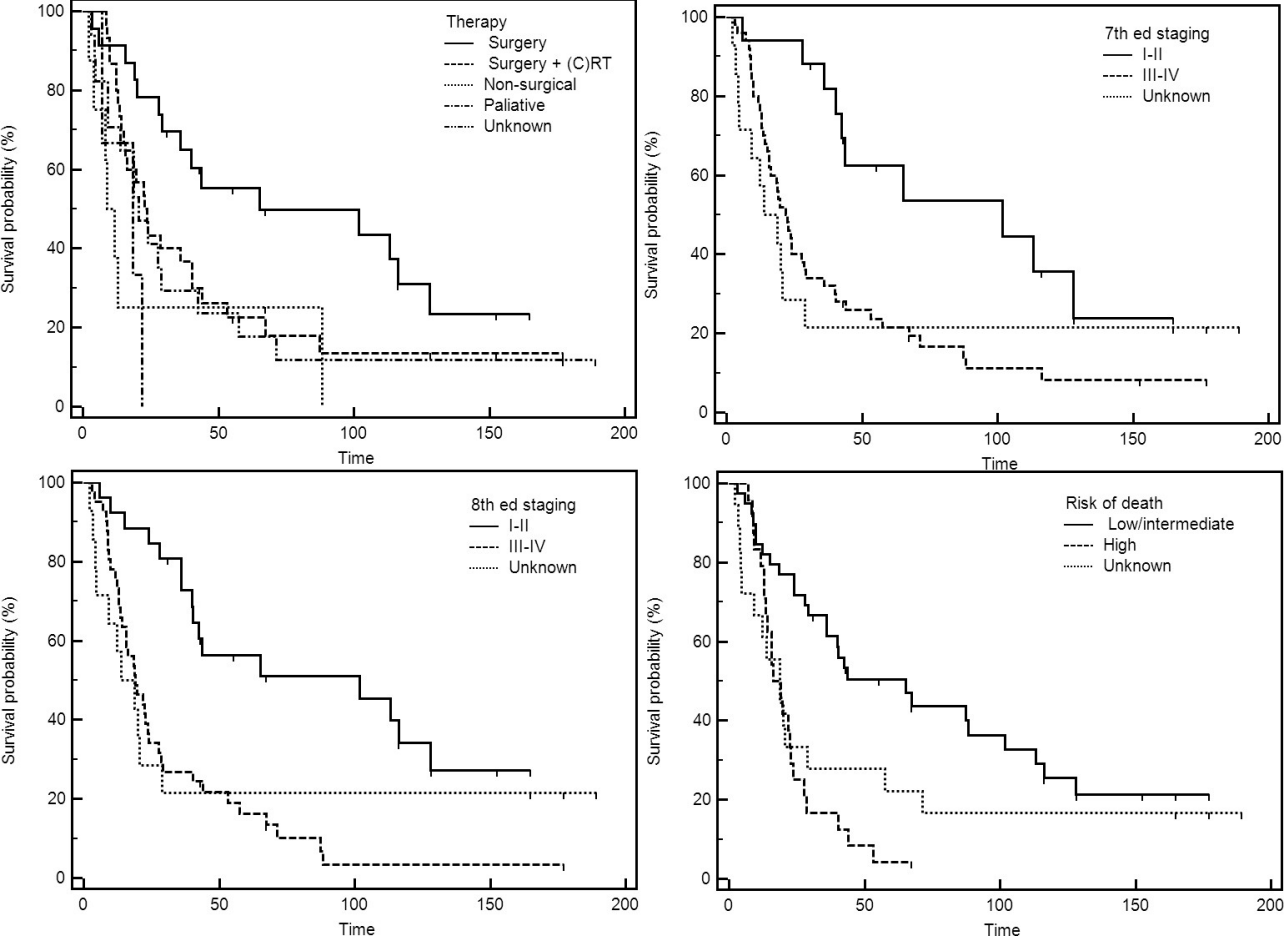
Overall survival of OPSCC patients based type of therapy, on 7th edition and 8th edition of staging guidelines and combined risk level (HPV status and T/N classification) based on those proposed by Ang et al. [24].

For survival analyses, 2 HPV RNA positive and 6 HPV RNA negative samples have been excluded, due to samples originating from recurrent and not primary tumors. In addition, for 10 patients, follow-up or survival data couldn’t be obtained, and have been excluded. Thus, all-cause mortality outcomes could be assessed for a total of 81 patients. Median follow-up (up to December 31^st^, 2017) was 23.9 months. The overall survival at 5-years was 30.9%. Kaplan-Meier survival curves for patient characteristics, clinical parameters, therapy and different staging approaches are presented in Figs 2, 3 and 4, respectively. Combined risk level, taking into account different patient aspects was aggregated and calculated as suggested by Ang et al [27]. The original method included the grouping by HPV status, smoking and T and N stages for HPV+ and HPV–cases, respectively. However, smoking was disregarded to retain more cases, as smoking information was not available for all patients. Moreover, low and intermediate groups were combined as there were only 5 patients satisfying “low risk” criteria based on the HPV RNA presence (Fig 4).

Patient characteristics (Fig 2) were not significantly (logrank test of Kaplan Myer survival curves) affecting survival even though female gender, younger age and no history of smoking/drinking were somewhat beneficial. Of clinical parameters (Fig 3), only T (*P* = 0.041) and N (*P* = 0.021) classification significantly affected the overall outcome. On the other hand, HPV+ and HPV–patients, regardless of the method of HPV detection (DNA, RNA or p16), had very similar survival. The effect of therapy on survival (Fig 4) was also significant (*P* = 0.019). In addition, both 7^th^ (*P* = 0.017) and 8^th^ (*P =* 0.001) editions of AJCC guidelines statistically significantly affected survival. The analyses based on combined risk level (HPV status and T/N stage) also significantly separated patients’ survival (*P* = 0.001). Cox proportional hazards regression in univariate and multivariate analysis are presented in Tables 3 and 4.

**Table 3:** Univariate proportional hazard regression of patient survival for tested variables.

Variable	Factor^a^	N	Relative risk	95% confidence interval	*P*	Overall model significance
Gender	F	16	1			P = 0.207
	M	65	1.5	0.7 to 2.9	0.229	
Age	continuous	81	1	0.9 to 1.1	0.063	P = 0.062
Age	<45	6				P = 0.127
	45–64	51	2.8	0.9 to 9.2	0.081	
	65+	24	2.6	0.8 to 8.9	0.126	
**T**	1–2	34	1			**P = 0.038**
	**3–4**	**29**	**1.9**	**1.1 to 3.5**	**0.017**	
	Unknown	18	1.8	0.9 to 3.4	0.079	
**N**	0–1	30	1			**P = 0.006**
	**2–3**	**33**	**2.4**	**1.4 to 4.3**	**0.003**	
	**Unknown**	**18**	**2.1**	**1.1 to 3.9**	**0.037**	
**Stage 7th**	I-II	17	1			**P = 0.009**
	**III-IV**	**50**	**2.6**	**1.3 to 5.2**	**0.006**	
	**Unknown**	**14**	**2.6**	**1.1 to 6.1**	**0.031**	
**Stage 8th**	I-II	26	1			**P = 0.001**
	**III-IV**	**41**	**2.9**	**1.6 to 5.4**	**0.001**	
	**Unknown**	**14**	**2.4**	**1.1 to 5.1**	**0.029**	
Grade	1	19	1			P = 0.876
	2	29	1.1	0.6 to 2.3	0.619	
	3	31	1.1	0.6 to 2.2	0.711	
	Unknown	2	2.4	0.5 to 10.7	0.246	
HPV overall	no HPV	49	1			P = 0.893
	inactive HPV	11	0.9	0.4 to 1.8	0.763	
	active HPV	21	1.1	0.6 to 1.9	0.776	
HPV DNA	no HPV	49	1			P = 0.964
	HPV DNA+	32	1.0	0.6 to 1.7	0.965	
HPV RNA	no HPV	60	1			P = 0.714
	HPV RNA+	21	1.1	0.6 to 1.9	0.712	
p16	p16 negative	36	1			P = 0.569
	p16 +	22	1.2	0.7 to 2.0	0.603	
	Unknown	23	0.8	0.4 to 1.5	0.510	
Alcohol	No alcohol	11	1			P = 0.075
	Occasional use	18	2.3	0.8 to 6.4	0.103	
	**Heavy use**	**8**	**4.0**	**1.3 to 12.3**	**0.015**	
	Unknown	44	2.5	0.9 to 6.3	0.059	
Smoking	No smoking	8	1			P = 0.279
	Former	9	2.7	0.7 to 10.3	0.138	
	Current	20	2.7	0.8 to 9.1	0.115	
	Unknown	44	2.7	0.8 to 8.7	0.099	

^a^Factors reaching statistical significance are highlighted by bold font

**Table 4:** Multivariate proportional hazard regression models for tested variables.

Variable	Factor^a^	Multivariate model 1 (n = 63)overall model significance P<0.001			Multivariate model 2 (n = 35)overall model significance P = 0.005		
		Relativerisk	95% confidenceinterval	*P*	Relativerisk	95% confidenceinterval	*P*
T	1–2	1			1		
	3–4	1.3	0.6 to 2.5	0.485	1.9	0.7 to 5.2	0.241
N	0–1	1			1		
	**2–3**	3.3	1.5 to 7.1	**0.003**	**4.6**	**1.2 to 17.0**	**0.023**
HPV overall	no HPV	1			1		
	**inactive HPV**	**2.3**	**1.0 to 5.0**	**0.043**	3.3	0.9 to 11.5	0.069
	active HPV	0.5	0.2 to 1.1	0.091	1.0	0.3 to 3.5	0.967
Alcohol	No alcohol	not included in model			1		
	Occasional use				1.4843	0.4 to 5.6	0.560
	Heavy use				2.9149	0.7 to 13.2	0.166
Smoking	No smoking	not included in model			1		
	Former				1.7463	0.3 to 9.1	0.510
	Current				0.8471	0.2 to 4.4	0.845

^a^Factors reaching statistical significance are highlighted by bold font

## Discussion

In this study, we performed HPV DNA and RNA analysis of oropharyngeal cancer patients treated within a 15-year period. The overall results indicate that 29.3% of cancer cases had active HPV E6 mRNA transcription and were thus likely HPV driven. The HPV DNA positivity, on the other hand, was shown in 40.4% patients, which is comparable with previous reports [13,16]. Previous studies [11,28–30] have already concluded that HPV DNA presence is not always sufficient to attribute cancer to HPV and that mRNA or combination of assays should be used. Unexpectedly, the correlation between p16 and HPV data was low with correlation coefficients r = -0.108 for comparison with HPV DNA and r = 0.146 for comparison with RNA suggesting that p16 is not a suitable replacement for HPV testing in the current setting. Some other studies have also shown that p16 testing might not be an accurate biomarker for oropharyngeal cancers, since the presence of p16 has been detected in HPV–HNSCC [31]. However, literature also suggests that p16 positive, but HPV negative HNSCC, share some common characteristics, like favorable prognosis, with HPV+ HNSCC [5,30,31]. However, in our study there were also several HPV+ yet p16 negative tumors that negatively affected the correlation. Such samples were also seen in the study of Jordan et al. [32]. However, recent Cancer Genome Atlas project analysis [33] indicated that CDKN2A gene (encoding p16 protein) is often deleted or mutated in smoking related HNSCC cancers. Such deletions would preclude p16 overexpression even if HPV is transcriptionally active.

This study encompass samples mostly from male patients with cancer in the oropharyngeal region, particularly base of tongue and tonsil, which is consistent with previous reports [13,34]. Those two regions also had the most HPV+ cases, which is in line with the literature [35] and cancers at those particular sites are the most responsible for the rising incidence of HNSCC in many Western countries [12,36,37].

Surprisingly, there were no significant differences in patient or clinical parameters between groups based on HPV RNA or only DNA or even p16 results. The overall 5-year survival was relatively low (30.9%), however, this was influenced by the lack of disease specific mortality data, which allows the calculation based only on all-cause mortality for the majority of patients. The use of all-cause mortality might also be masking the effects of other variables, which, with the exception of T and N stages (*P* = 0.041 and *P* = 0.022, respectively), failed to adequately stratify patient risk of death on Kaplan-Myer analysis. However, the combined risk stratification as proposed by Ang et al [27] originally combining HPV, smoking (not included in our calculation) and TNM stage almost perfectly classified patients in low/intermediate and high risk of death groups (Fig 4; *P* = 0.001). Similar results were obtained on univariate and multivariate Cox proportional hazards regression (Tables 3 and 4). Namely, only T and N classifications (and staging based on this variables) significantly affected survival with the N classification significant in both univariate and multivariate models. However, HPV did not confer significant survival benefit in either test.

The lack of impact of HPV or even p16 on survival was surprising; however, several factors could be responsible. One of the major influences that could mask any positive HPV/p16 effects on survival is smoking and drinking history. Only 14% of patients never smoked and 13% never consumed alcohol with the majority still being an active smoker and at least moderate drinker (Table 1). Smoking is still a significant problem in Croatia [38], with almost no change in smoking prevalence seen for males in the 1994–2005 period with a slight decrease from 34.1% to 33.8%. The more recent World Health Organization (WHO) report on smoking prevalence [39] estimates an even greater male smoking prevalence in Croatia for year 2015 at 37.9% with the similar rate, around 24% for UK or USA. This, together with previous studies that have shown that smoking can have the greatest effect on survival and even outweigh HPV effect [40,41], most probably explains our findings. The hazardous effect of smoking on cancer risk was shown for both HPV+ and HPV–HNSCC [42] even at low doses [43]. Furthermore, the strong influence of smoking on cancer in Croatia is also demonstrated in a recent review of lung cancer [44]. Therein, Croatia was among 20 countries with the highest incidence of lung cancer in the world. Thus, Croatian patients are yet to benefit from smoking cessation programs and past smoking history most likely influenced development of associated cancer types. It was moderately surprising that smoking was not significantly affecting survival on Cox regression analysis; however, it is likely that patients self-reported less smoking than they actually did due to the recent social stigma associated with smoking and antismoking campaign. The multivariate models also suffer from lack of data since only 35 patients had complete information for analysis. Similarly, alcohol drinking is known to increase cancer risk [45] and it is prominent in the Croatian population. The WHO report on alcohol consumption [46] put Croatia among the top countries according to age standardized alcohol-attributable malignant neoplasm death rates in 2016. Interestingly the univariate model indicated that heavy drinking (and/or strong spirits use) significantly decreased survival (Table 3) indicating that alcohol might represent even a higher health concern than smoking in Croatia.

Another factor potentially influencing our findings is that, surprisingly, there were no significant differences between groups with regards to age at diagnosis in our study population, which might also hint at a more classical etiology. Previous studies have shown that HPV+ HNSCC primarily affects younger patients with higher socio-economic status and this particular group is the underlying cause for increasing incidence of HNSCC [5,34,47–49]. It appears that in Croatia, and possibly elsewhere, societal and life style changes are lagging behind more developed countries and not enough time has elapsed to shift the importance of risk factors in HNSCC development from classical smoking and drinking to the new HPV related risks. There appears to be no significant shift in incidence (particularly in younger patients) or survival usually associated with HPV positivity. Interestingly, the study by Nygard et al [49] showed that in Norway, there was a period (1981–1995) in which the survival of HPV+ HNSCC was even worse than the HPV–HNSCC. However, in more recent years (1996–2007) there was a dramatic shift in the survival of HPV+ patients. The median age of diagnosis decreased from 63.2 to 59.8 years for HPV+ but remained unchanged at 66 for HPV–patients. In our study, there was no observable difference in age at diagnosis between patient groups, and there were no survival differences, thus, making the overall results comparable to the 1981–1995 period in Norway. This indicates that in our non-Western population additional, classic, factors are somewhat more involved in HNSCC outcomes. On the other hand, some of the “younger population” trends were recently shown to be changing in the Western populations as well [6,7]. However, data [8] also suggests that the prognostic advantage of HPV is attenuated in older patients.

Current literature also suggests significant geographical variations of HPV involvement in HNSCC. This was recently supported by a study [50] analyzing data from 4 multinational randomized trials, which suggested geographic variability as being important for OPSCC. In the study of Mehanna et al., HPV+ (DNA and p16 together) HNSCC seemed to be of less relevance in Eastern Europe (6%) and Asia (2%) compared to more developed Western countries (37%), where most of current literature derives from [50]. For comparison, in our study, there were only 8 cases where both DNA, RNA and p16 were positive (7%) or 10 cases (8.7%) where only DNA and p16 were positive, suggesting that the data is similar and HPV doesn’t play a major role in HNSCC in this geographical region. Similar data is seen in other socio-economically alike populations as Spain [51], where HPV/p16 positive samples amount to 6% of OPSCC cases and there was no significant survival improvement for HPV/p16 positive cases. Data from geographically closer Italy suggests that HPV driven HNSCC there are also underrepresented compared to the rest of Western/Northern Europe. However, survival was positively affected in their HPV driven tumor patients [52], which might also be affected by relatively greater economic development of North Italy region analyzed therein.

Another recent study describes predictors of OPSCC survival in Europe [53]; the analysis was based on 321 OPSCC subset with similar patient characteristics as ours. The study indicated reduced hazard ratio of 0.59 for HPV serology positive patients, whereas, in our study there were no significant differences in survival, which is most likely due to potential behavior differences between Western and non-Western populations.

Our study had several limitations. The first limitation was due to the nature of FFPE samples where the nucleic acid quality is suboptimal, however, this was mitigated by the use of PCR assays suitable both for DNA and RNA amplification from FFPE material. Furthermore, internal control beta-actin amplifications were performed to assess material suitability before HPV amplification. The second limitation was the retrospective nature of the study, where proper documentation was not always available; sometimes detailed follow-up was impossible because the patient visited other hospitals after initial treatment. Therefore, the medical records were supplemented by data (if available) from the Croatian Cancer Registry that on a national level collects relevant information on patient’s cancer irrespective of where the patient was referred, and also contains survival data. The European multicenter study [53] also faced data obstacles to a similar extent (22% missing cancer stage information herein; 16% in their study). Finally, the third limitation was the relatively small size that correlates with the incidence of oropharyngeal cancer in Croatia. However, to put our results in to context, the recent meta-analysis by Albers et al [30] looking at the influence of HPV and p16 patterns on HNSCC patient survival included 25 different studies of which the current study falls roughly in the middle of regarding sample size.

## Conclusions

In summary, this study provides the baseline relevant data for treatment of OPSCC patients in Croatia. Eventual policy and treatment decisions in similar regions should take into account the particularities of each population. Other factors like advanced stage, patient age or still highly prevalent smoking and drinking in Croatia might be overshadowing the positive effect of HPV seen in Western populations. Current data indicates that HPV, as a favorable prognostic marker, should not be considered to outweigh other relevant factors in a particular population until other socio-epidemiological changes evident in Western populations are also observed.
